# Domain learning naming game for color categorization

**DOI:** 10.1371/journal.pone.0188164

**Published:** 2017-11-14

**Authors:** Doujie Li, Zhongyan Fan, Wallace K. S. Tang

**Affiliations:** Department of Electronic Engineering, City University of Hong Kong, Kowloon, Hong Kong; Zhejiang University, CHINA

## Abstract

Naming game simulates the evolution of vocabulary in a population of agents. Through pairwise interactions in the games, agents acquire a set of vocabulary in their memory for object naming. The existing model confines to a one-to-one mapping between a name and an object. Focus is usually put onto name consensus in the population rather than knowledge learning in agents, and hence simple learning model is usually adopted. However, the cognition system of human being is much more complex and knowledge is usually presented in a complicated form. Therefore, in this work, we extend the agent learning model and design a new game to incorporate domain learning, which is essential for more complicated form of knowledge. In particular, we demonstrate the evolution of color categorization and naming in a population of agents. We incorporate the human perceptive model into the agents and introduce two new concepts, namely subjective perception and subliminal stimulation, in domain learning. Simulation results show that, even without any supervision or pre-requisition, a consensus of a color naming system can be reached in a population solely via the interactions. Our work confirms the importance of society interactions in color categorization, which is a long debate topic in human cognition. Moreover, our work also demonstrates the possibility of cognitive system development in autonomous intelligent agents.

## Introduction

Naming game (NG) model describes the interaction of two agents, targeting for knowledge transfer [1]. In a typical NG, the first agent (the speaker) will speak out the name of an object and ask the second agent (the hearer) to point out the named object amongst several objects. If the hearer is correct, the game succeeds. Otherwise, it fails, and the speaker will indicate the corrected object to the hearer so that the hearer can learn and update its memory.

NG can be simplified by without referring to any object, which is now commonly called as minimal naming game (MNG) [2]. In a typical MNG, the speaker picks a name randomly from its memory, and if the hearer also has the same name in the memory, it is a success game. Both of them will only keep this name and delete all the others from the memory. If it is a failure, the name will be added to the hearer’s memory.

NG and MNG have been considered under a complex network framework, where agents are represented by nodes of the network and games are conducted between connected agents. Networked NG and MNG are useful in studying the learning dynamics in a population of agents, where the reaching of consensus is the key objective. A lot of research work have been carried out to study how the agent selection process, both for the speaker and the hearer, may affect the reaching of consensus and the success rate of the games [3–8]. The impacts of topological features of the underlying network, such as clustering, regularity, distance and degree, have also been investigated [9–11].

Agents in a population are not necessarily identical but may possess different propensities. The studies in [12, 13] considered agents with high commitment and [7, 14] investigated agents with stubbornness. The inclusion of these agents affects the learning of agents, which in turn, brings a significant impact to the reaching of consensus. Other factors that reflect the real situation in learning have also been investigated. In [15], memory loss in agents is implemented. Hence, an agent may forget some words in its memory but it keeps the transmitted words unchanged. In [16], learning errors were considered so that the conveyed word could be replaced. A bidirectional way of learning is suggested for MNG in [17]. If it is a success, all names common in both the speaker and the hearer are kept. Otherwise, both of them update their memories by merging with the other’s.

It is remarked that most of the previous researches only focus on the emergence of language [8, 18, 19]. It is not limited to the vocabulary development and naming objects, but can be used for other linguistic feature, for example, the grammatical agreement game model in [20]. However, existing studies put emphasis on language development, but ignore the corresponding conceptual learning involved in object naming. When a new concept is acquired, we not only just remember the name but also characterize the features of the object. For example, when we refer to “a car”, we identify it with the features, such as four wheels, engine, carrying persons, etc. Confronting with these realistic cases, the current NG models would be inadequate and therefore a more complicated model is urged.

In this paper, we are particularly interested in color naming and categorization, which is one of the important cognitive developments for human being. There have been long debates between relativist and universalist in this topic. On one hand, the universalist argues that there are some universal focal colors due to the pan-human physiological processing of color stimuli. This view is supported by the findings that color memory appeared to be privileged for these colors, even in largely different color language systems [21–23]. On the other hand, the view of relativist states that color processing is relied on the learned language associations and perceptual learning specific to a given culture [18, 24, 25]. It can be noticed that, in either case, the impacts of the learning process in the society are seldom taken into account.

There is limited work in game design focusing on color categorization. In [25], color categorization was studied based on two types of games, called discrimination game and guessing game. In a discrimination game, a set of color samples is used to train the speaker’s classifier network so that the samples can be classified into different names. After training, guessing game is employed. Guessing game is similar to NG, except that the hearer will update the classifier network. It must be remarked that a pre-requisition is needed, i.e. the color samples must be different in classification. Another specific game for categorization was proposed in [26], known as Category Game Model (CGM). Before a game, the speaker is required to distinguish two objects provided to both players. If the two objects are indistinguishable to the speaker, a new category is generated. Then, the speaker assigns one of these objects to teach the hearer, which is identical to guessing game in [25]. The CGM has been further studied in [27, 28]. However, a “God” is always required to provide two different objects to the agents.

We here question whether it is possible for agents to autonomously develop a color naming system together with the category of colors solely based on interactions, without any pre-requisition in the learning process. As mentioned in [29], the challenge is that the agents have to develop a mapping from a continuous domain to a finite and discrete number of color categories. In [29], Euclidean distance is assumed, which unfortunately fails to match with human perception. In this work, we propose a new learning process, called Domain Learning Naming Game, based on the understanding of human perception and cognition. Two processes in color categorization, namely color perception and color naming, are incorporated in agents. As demonstrated by simulations, agents are able to develop a consensus in color learning system with the color domains specified by names. Some interesting observations are also reported.

## Methods

### Color space

The domain learning naming game (DLNG) is conducted over a group of networked agents, who can detect colors in the CIELab color space. Each color is represented by a triplet (L*, a*, b*) where L* stands for the lightness, a* and b* are the two opponent dimensions [30]. CIELab is preferred in our model because its design is to approximate human vision. The range of L*, a* and b* are set as [0,100], [-100, 100] and [-100, 100], respectively, in our study, covering nearly all the RGB colors. The color space is further divided into 25×50×50 sub-spaces, each is a cube with 4 units in each of the three dimensions. Ω is a set containing the centroids of all the sub-spaces where |Ω| = 62500 is the cardinality of the set Ω.

Human perception on color is a subjective process. If two colors are too close, they are not distinguishable. The farness of two colors can be specified by a distance metric called Delta-E (Δ*E*) in the CIELab color space. The most recent CIE2000 [31, 32] is adopted in this project because (i) it is commutative, i.e. Δ*E* (color 1, color2) = Δ*E* (color 2, color1) [Note: Commutative is not always true for some versions, eg. CIE94]; (ii) it is close to the color discrimination of human eye. Table 1 shows the relationship between human cognition and the value of ΔE [33].

**Table 1:** ΔE and the corresponding cognition.

ΔE	Cognition
ΔE≤1	Imperceptible by human eyes
1<ΔE≤2	Cognizable by close observation
2<ΔE≤10	Cognizable at a glance
10<ΔE≤50	Clear difference in colors is noticed
ΔE> 50	Exact two different colors

**Fig 1:**
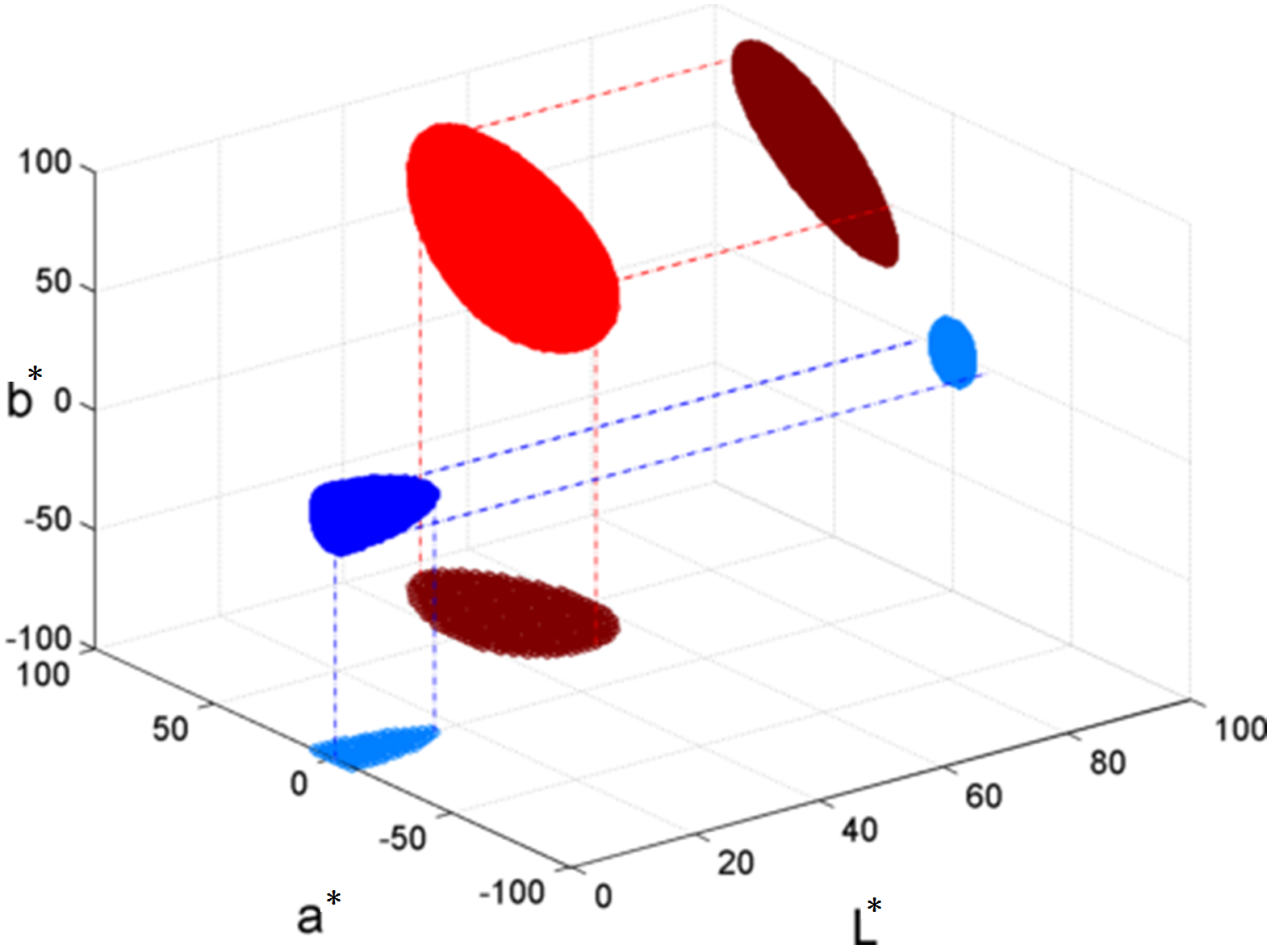
RED oval indicates colors that have ΔE≤10 with color (0,0,0); BLUE oval indicates colors that have ΔE≤10 with color (50,50,50), based on CIE2000. Their projections onto L*–a* and a*–b* planes are shown in BROWN and LIGHT BLUE, respectively.

It should be remarked that ΔE is a nonlinear function. Consider an example in Fig 1, colors that have ΔE≤10 with color (0,0,0) and color (50,50,50) are marked in red and blue, respectively. Their corresponding projections onto the L*–a* and a*–b* planes are shown, clearly demonstrating the non-uniformity and color-dependence of the function.

In a game, only the color of the centroids in Ω is considered and the color of each centroid may be associated with at most one name. Initially, agent has no knowledge of any color, so there is no name associated to any centroid.

### Agent design

The color perception of an agent involves two important concepts, namely subjective perception and subliminal stimulation [34, 35]. Subjective perception is related to one’s viewpoint. If ΔE of two colors is too large, say larger than a threshold *SP*_*T*_, these two colors are definitely not the same. On the other hand, subliminal stimulation refers to the sensory stimulation, and colors are non-differentiable if their Δ*E* is too small, say smaller than or equal to a threshold *SS*_*T*_. It thus reflects the sensitive of the cone-cells of an agent, and obviously, *SS*_*T*_ *< SP*_*T*_.

In our game, when a color *C*_*i*_ ∈ Ω is perceived, two corresponding sets Ω_*SP*_(*C*_*i*_)and Ω_*SS*_(*C*_*i*_) are formed in the agent’s memory:

$$\Omega_{SP}(C_{i})=\{C_{j}\in \Omega |\Delta E(C_{i},C_{j})\leq SP_{T}\}$$ (1)

$$\Omega_{SS}(C_{i})=\{C_{j}\in \Omega |\Delta E(C_{i},C_{j})\leq SS_{T}\}$$ (2)

**Fig 2:**
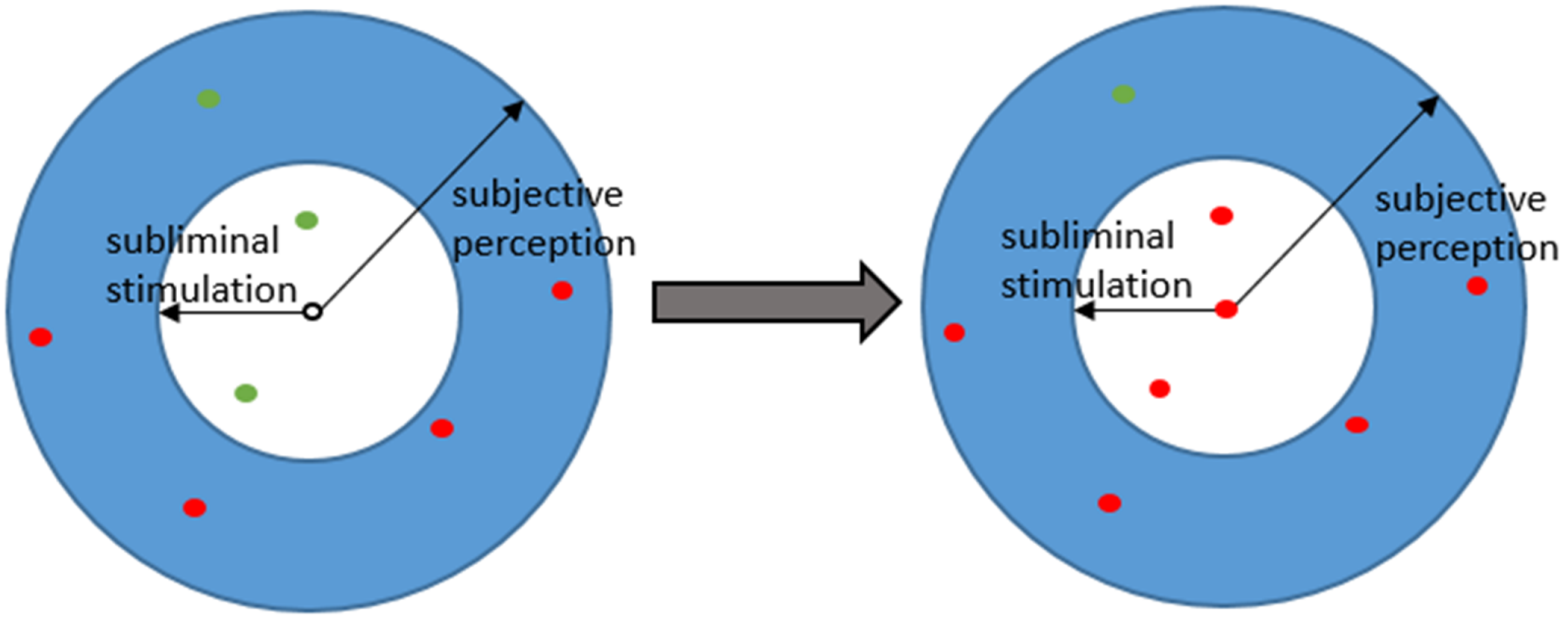
Perceptive model of an agent and color memory update.

The color *C*_*i*_ is then classified by following the majority rule over the names in Ω_*SP*_(*C*_*i*_). Considering an example depicted in Fig 2, eight color centroids belong to Ω_*SP*_(*C*_*i*_), where four of them are named as “red”, three of them are named “green” and *C*_*i*_ itself is unnamed. Based on the majority rule, *C*_*i*_ is then named as “red”. Also, according to subliminal stimulation, all the color centroids belonging to Ω_*SS*_(*C*_*i*_) will be named as “red” consequently (see Fig 2).

### Design of the game

The domain learning naming game is conducted in a population of agents with connectivity defined by an underlying topology, where each agent is as described before. The procedures of the game are given as follows:

1. An agent is randomly selected from the population and it acts as the Speaker.
2. One of the Speaker’s neighbors is randomly selected as the Hearer.
3. A color is randomly selected from the set Ω, say *C*_*i*_ ∈ Ω.
4. The Speaker will get the name of *C*_*i*_ following the perceptive model explained in Fig 2. If there are more than one name having the same highest occurrence frequency in the set Ω_*SP*_(*C*_*i*_), the Speaker will randomly choose one of them. On the other hand, if there is no name in Ω_*SP*_(*C*_*i*_), a new name will be generated. Finally,*C*_*i*_ is categorized, say as X.
5. The Speaker will then update its memory by assigning X to all *C*_*x*_ with *C*_*x*_ ∈ Ω_*SS*_(*C*_*i*_).
6. The Hearer will follow the same step as in Step 4 to categorize the color *C*_*i*_.
7. If the names from the Speaker and the Hearer are the same, the game succeeds. The Hearer will then assign the name X to all *C*_*x*_ with *C*_*x*_ ∈ Ω_*SS*_(*C*_*i*_).
8. If the names are different, the game fails. The Speaker will inform the Hearer the correct answer and the Hearer will update all *C*_*x*_ ∈ Ω_*SS*_(*C*_*i*_) with the correct name.

**Fig 3:**
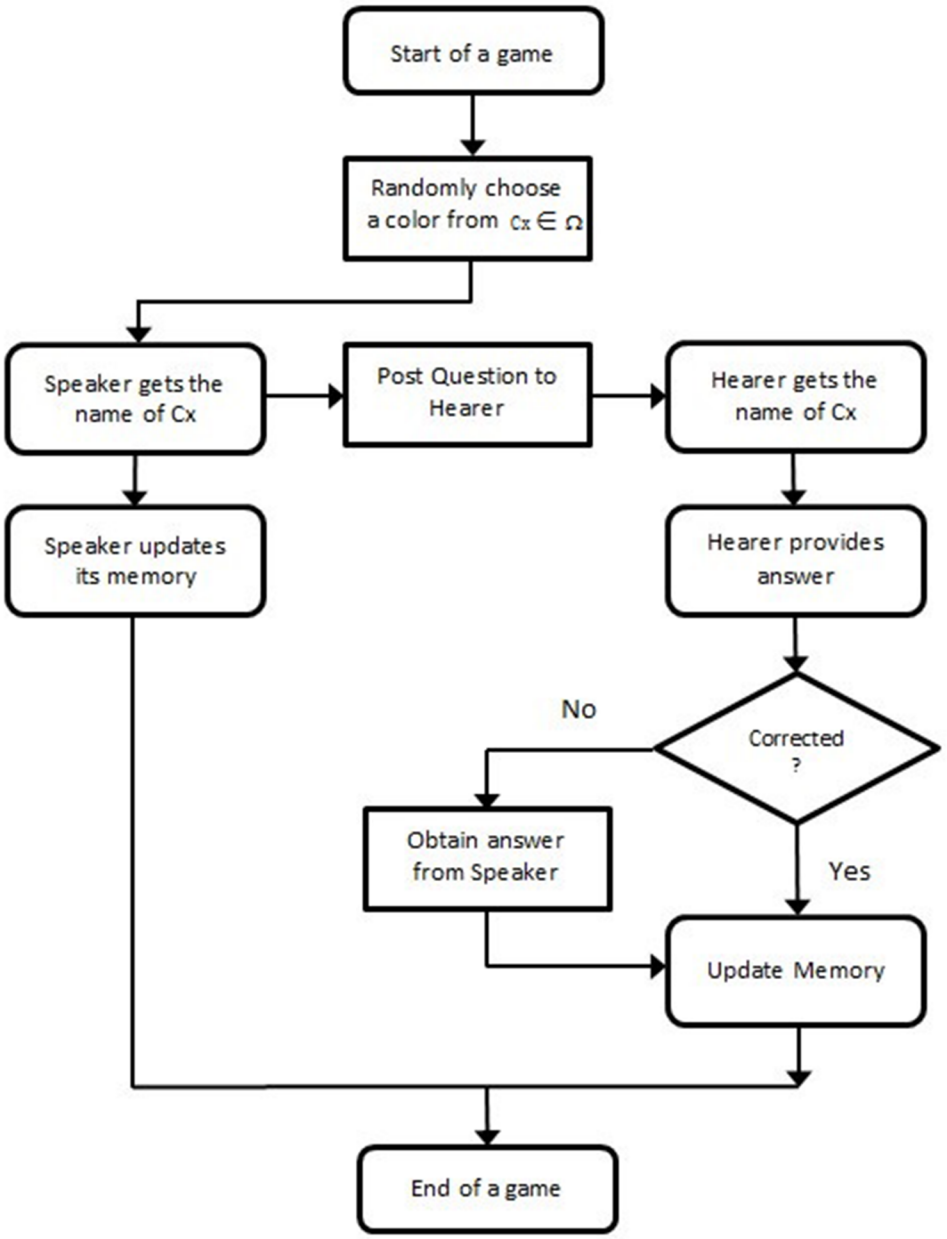
Flow diagram of DLNG.

The overall flow diagram of the game is depicted in Fig 3.

## Results

### Simulation setup and measurements

The domain learning naming game has been applied for two cases, with the number of agents equal to 100 and 300, respectively. In each case, the connections between agents are made according to the procedures specified by the BA scale-free model in [36], with an average degree of 4. To eliminate randomness, all the reported results in this paper are average of five independent runs.

Four different metrics related to the learning process are measured for every 10,000 games.

1. Accuracy of the agent (ACC): Accuracy is defined as the success rate of the game.
2. Number of unnamed centroids (UNC): This indicates how many color centroids have never been associated to any name.
3. Number of total different names in population (TDN): It records how many different names remain in agents’ memory during the learning process.
4. Number of different names in each agent (DN): It records the number of different names in each agent. The maximum, the minimum and the average of DNs are computed. It is to provide an overview on the learning performance.

Unlike the conventional game, ACC may not provide a good criterion for terminating the program. As explained in Agent Design, the name of a tested color is determined by the majority rule. Therefore, agents may maintain diversity even ACC is high.

Here, we propose to use TDN as the criterion. The program will first run for one-million iterations, where every iteration involves one game. Then, TDN is recorded for every 10,000 iterations. If the value of TDN keeps unaltered for the consecutive one million iterations, the program will be terminated.

Since the results are similar, we only include results for the network of 300 agents here, while for the case of 100 agents, it can be found in S1 Appendix.

### Baseline results

**Fig 4:**
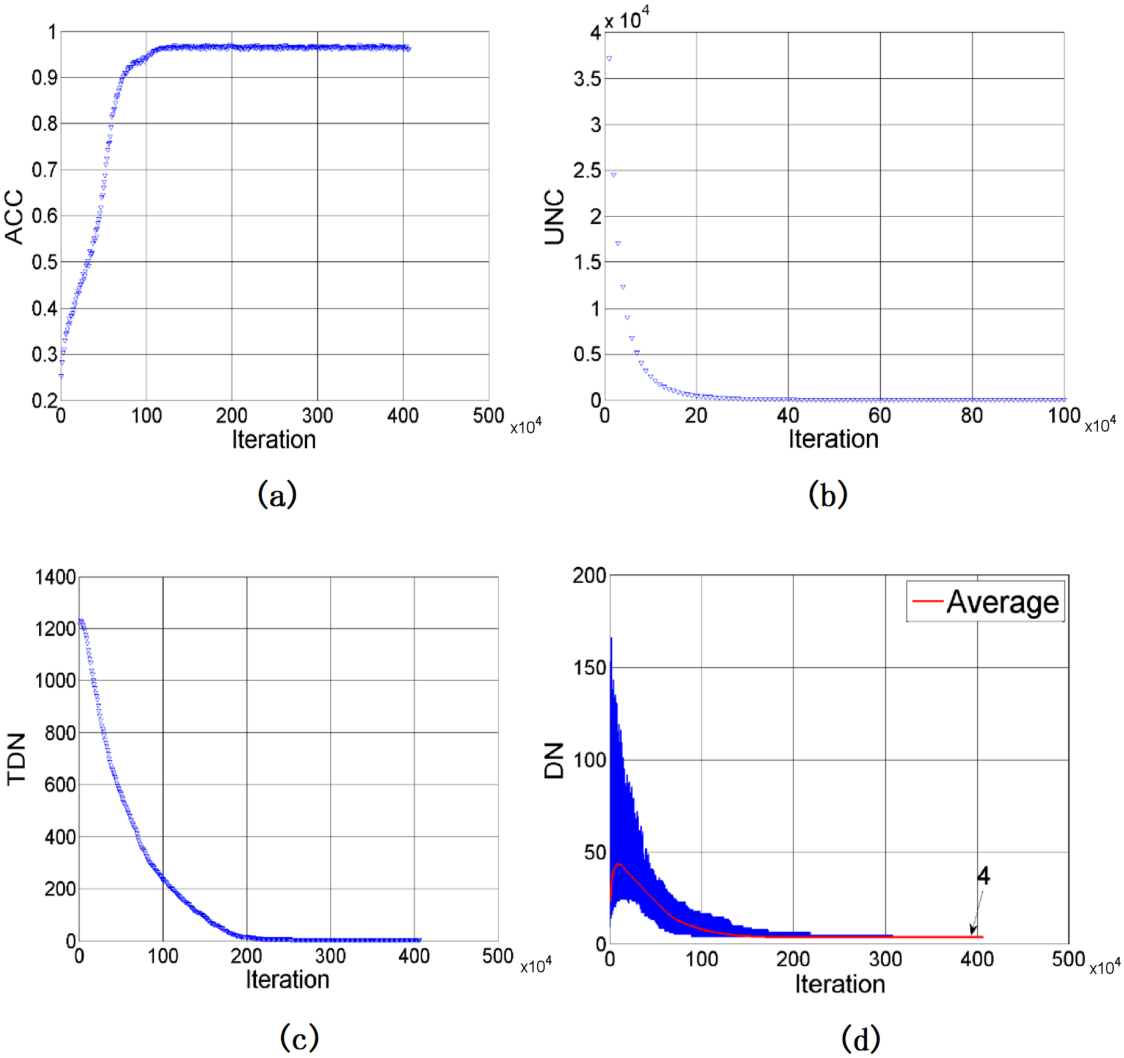
Simulation results of DLNG with *SP*_*T*_ = 20 and *SS*_*T*_ = 10: (a) ACC, (b) UNC, (c) TDN, (d) DN.

Fig 4 depicts the baseline results by letting *SP*_*T*_ = 20 and *SS*_*T*_ = 10. Since there is no name in the agents initially, ACC starts with zero. When agents learn from each other via interactions, colors are categorized with names and the chance having agreement in color’s name between agents in a game increases. This not only results in a sharp reduction of UNC but also a gradually increase of ACC. As shown in Fig 4(b), UNC becomes zero in about 1,000,000 games. However, the way to categorize color may be different amongst agents. Being interacted in more games, ACC gradually increases and finally reaches 96.97% after 4,070,000 games. The value of TDN confirms this observation from another view. The TDN increases sharply similar to UNC, but it gradually decreases due to the learning, matching with the trend in ACC, and eventually reaches a very small number which is four. Fig 4(d) depicts the ranges of DN of agents in the population. The value of DN varies from 9 to 153 initially, but these names are largely different as we have a large TDN. By participating in games, both variation and average of DNs drop as shown in blue and red, respectively, in Fig 4(d). Finally, the entire color space is categorized into four types of colors.

**Fig 5:**
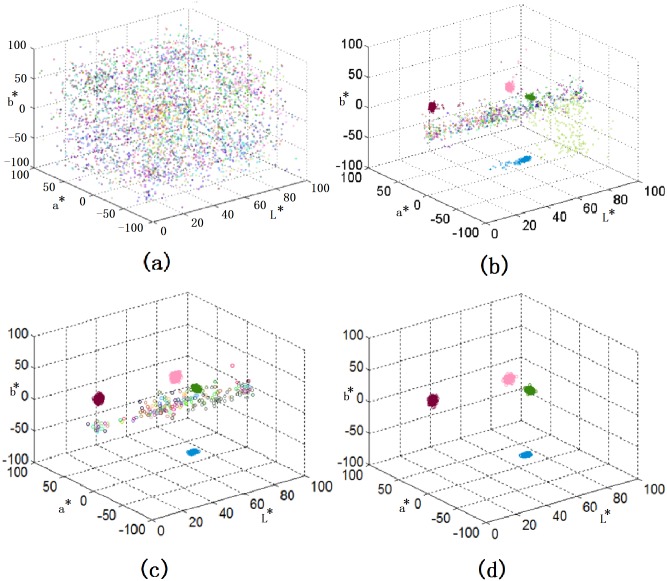
Variation of memory in the agents (a) after 100,000 games, (b) after 1,000,000 games, (c) after 1,500,000 games, (d) after 4,000,000 games.

**Fig 6:**
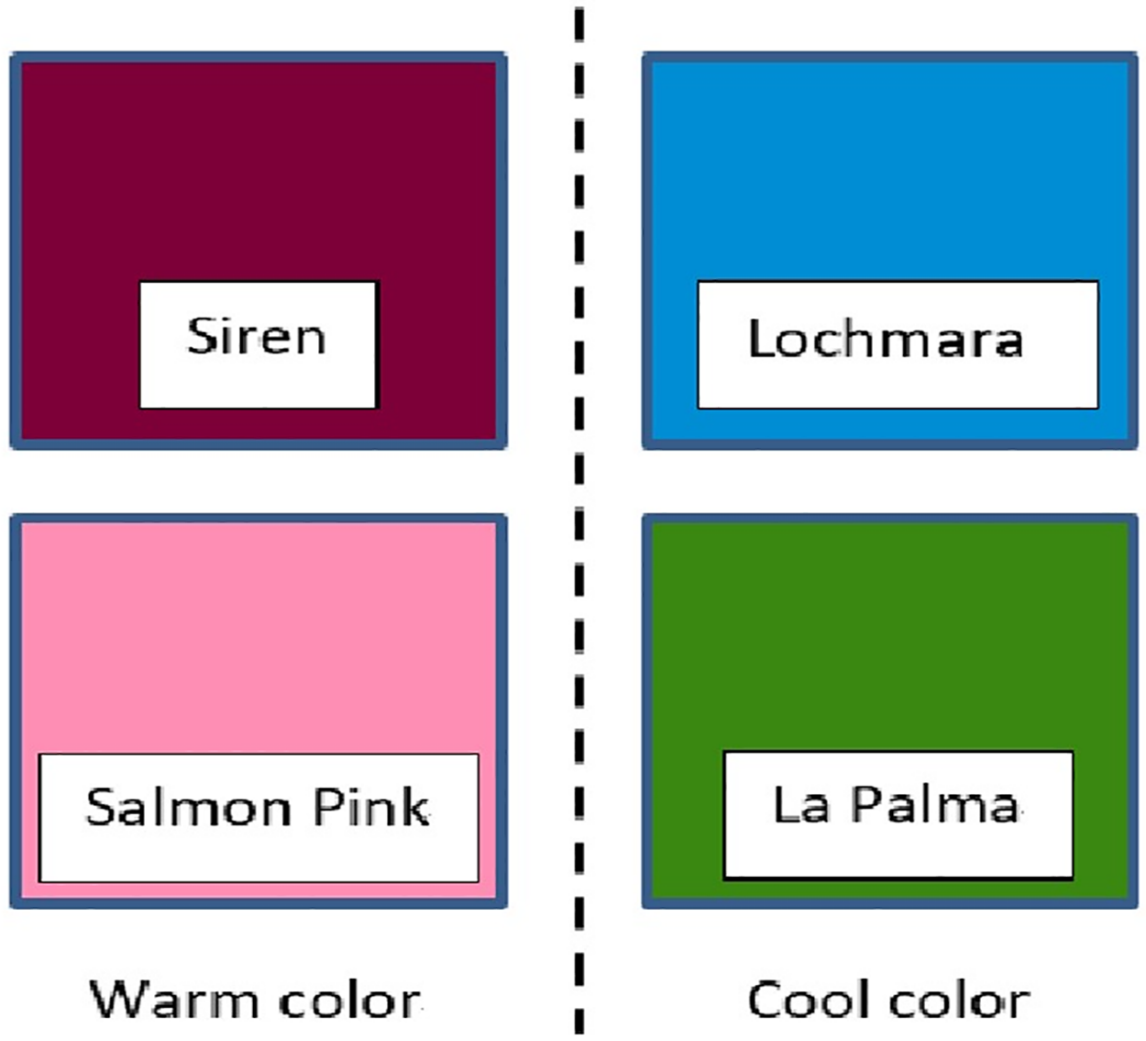
Actual colors for the mean (L*, a*, b*) of color centroids specified by the final four names.

To illustrate how agents’ memories evolve during the learning process, we plot the names in all 300 agents in Fig 5. Each dot indicates the mean coordinates of the color centroids having the same name, and different colored dots are assigned to different names. It can be observed from the evolution in Fig 5, consensus of color categorization can be reached and finally there are only four names in each agent as shown in Fig 5(d). It is interesting to notice that the obtained color categories are close to the basic colors reported by Berlin and Kay [22] in their seminal work on color languages. Based on their study and many others, it is now commonly agreed that there are 2 to 12 basic color terms [37]. Besides BLACK and WHITE, the colors RED, BLUE, GREEN and YELLOW are high in the color hierarchy. In our results, we obtain SIREN (a kind of RED), LOCHMARA (a kind of BLUE), and LA PALMA (a kind of GREEN) represented by the mean coordinates of the three names (see Fig 6). Moreover, SIREN and the fourth color SALMON PINK help to distinguish the lightness of colors, similar to the contrast in BLACK and WHITE for color naming. Indeed, our results also seem to follow the partition rules in [37], which suggest that color partitions are based on (i) BLACK and WHITE, (ii) warm and cool primaries, (iii) distinguish of RED. In our results, SIREN and SALMON PINK are the warm colors, while LOCHMARA and LA PALMA are the cool colors, though they are not the primaries. Moreover, the obtained SIREN and SALMON are not only good to distinguish lightness of colors but also provide a more detailed in distinguishing RED colors.

From Fig 5(d), it is also observed that the categorizations in different agents are not identical but close. That is different from conventional naming games, in which agents are identical in consensus. However, it is common in color naming experiments. Human participants with same language group may still have different results in color categorization, for example those reported in [38]

### Impacts of subliminal stimulation

**Fig 7:**
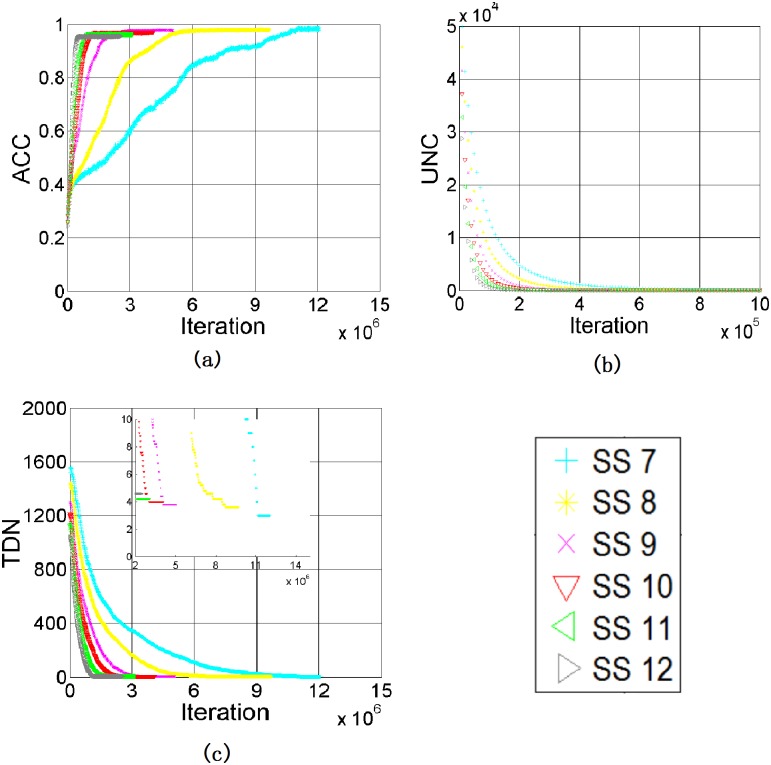
Results with *SP*_*T*_ = 20 and different values of *SS*_*T*_: (a) ACC, (b) UNC, (c) TDN.

We here investigate the impacts of subliminal stimulation onto the evolution process of color categorization in agents. Fig 7(a)–7(c) plot ACC, UNC and TDN against iterations, respectively, and Table 2 lists their final values after 2,000,000 iterations.

**Table 2:** ACC, TDN and UNC for *SP*_*T*_ = 20 and different *SS*_*T*_ after 2 million iterations.

*SS*_*T*_	ACC	TDN	UNC
7	98.70%	3.0	0
8	98.00%	3.6	0
9	97.73%	3.8	0
10	96.97%	4.0	0
11	96.27%	4.2	0
12	95.48%	4.6	0

Based on the definition of *SS*_*T*_, if *SS*_*T*_ is larger, the agent considers a larger range of colors to be the same. Thus, more color centroids will be assigned with the same name in one game. Consequently, the learning rate increases as shown in Fig 7. However, it is interested to notice from Fig 7(a) and 7(c) that ACC reduces and TDN increases when *SS*_*T*_ increases. Recall that *SS*_*T*_ reflects the vision sensitive of agents, the larger *SS*_*T*_ is, the less sensitivity in color is (eg. the agents cannot distinguish different levels of red colors). In this case, agents would reach consensus easily but our results also show that the level of consensus drops.

**Fig 8:**
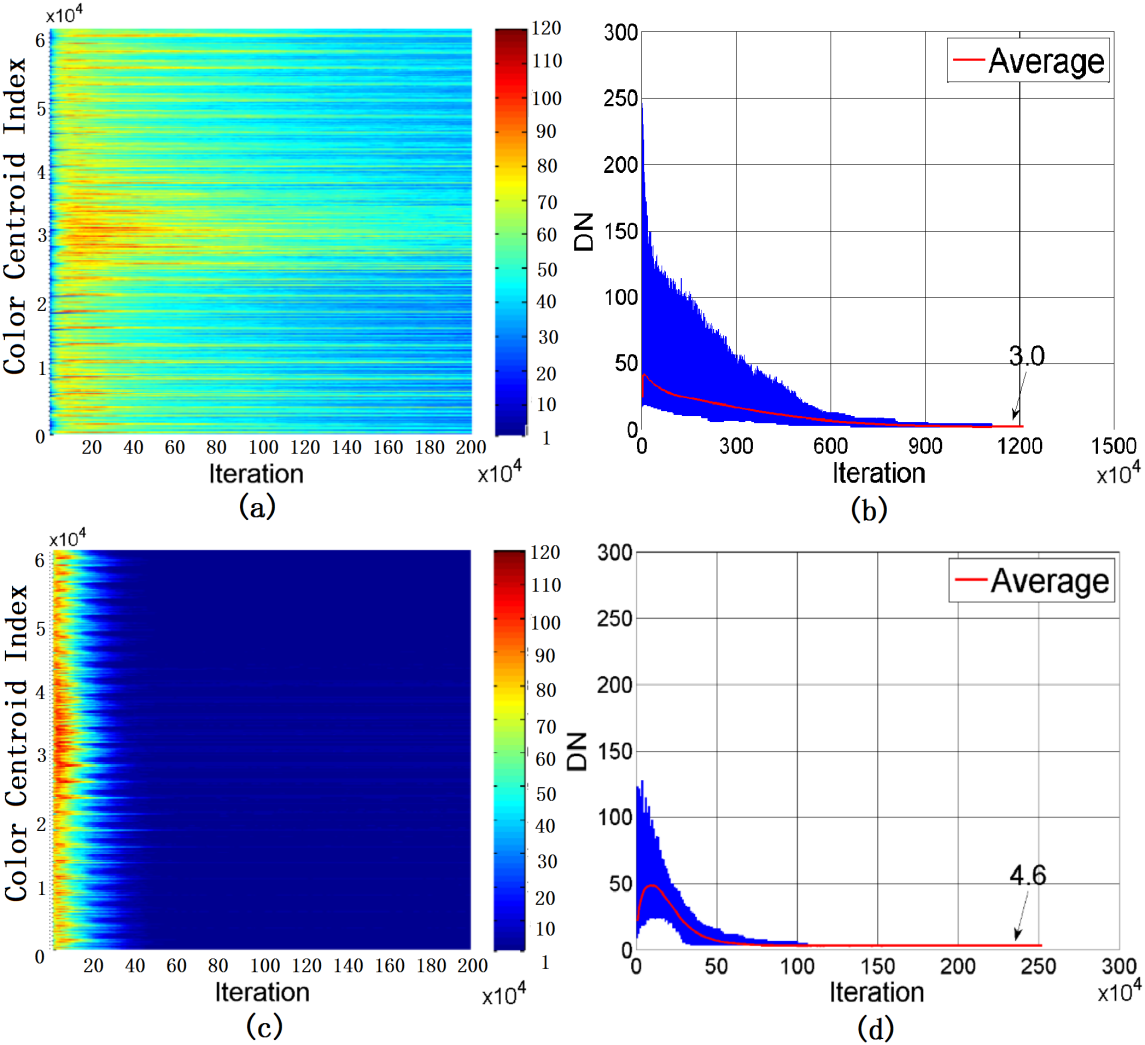
Evolution of the name variation for Ω: (a) and (b) for *SS*_*T*_ = 7 (c) and (d) for *SS*_*T*_ = 12.

**Fig 9:**
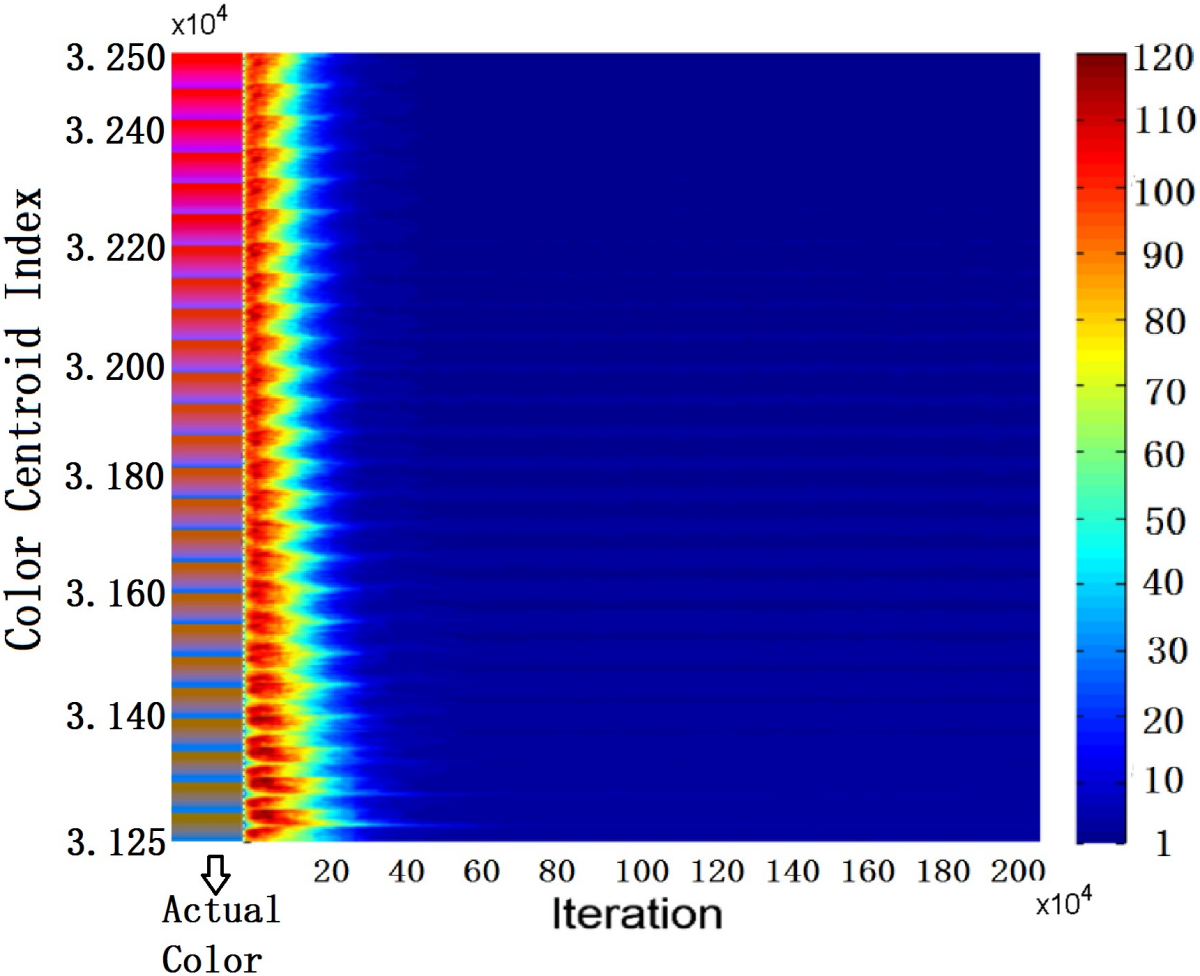
Evolution of the name variation for color centroids with L* = 50 for *SS*_*T*_ = 12.

To provide further details, we compare the variations of names in agents for *SS*_*T*_ = 7 and *SS*_*T*_ = 12, and the results are given in Fig 8. The color of each dot in Fig 8(a) and 8(c) indicates the total number of names for that particular color centroid. When *SS*_*T*_ is small, agent creates (as the speaker) or acquires (as the hearer) more names because it is relatively higher chance to have no name for a color centroid. The learning process is slower. Since the number of color centroids to be updated in each game is few, the number of different names is slowly reduced. Eventually, after a long time of interactions, there are only 3 names left in each agent, implying that the coverage of each name is indeed larger. In contrast, when *SS*_*T*_ is large, the accumulated number of different names in each agent is much fewer, the learning pace is faster, but there are average 4.6 names at the end with a worse ACC (see Table 2). It is also interesting to observe that the learning process of different colors varies. Fig 9 shows a zoomed version of Fig 8(c) with a fixed value of L* (L* = 50). By referring to the actual colors of the centroids, it can be concluded that it is more different to learn BLUE color than RED color. This observation has also been noticed in many other human involved experiments, supported by the fact that RED is higher in color hierarchy as defined in the Basic Color Terms Theory [22].

### Impacts of Subjective Perception

**Fig 10:**
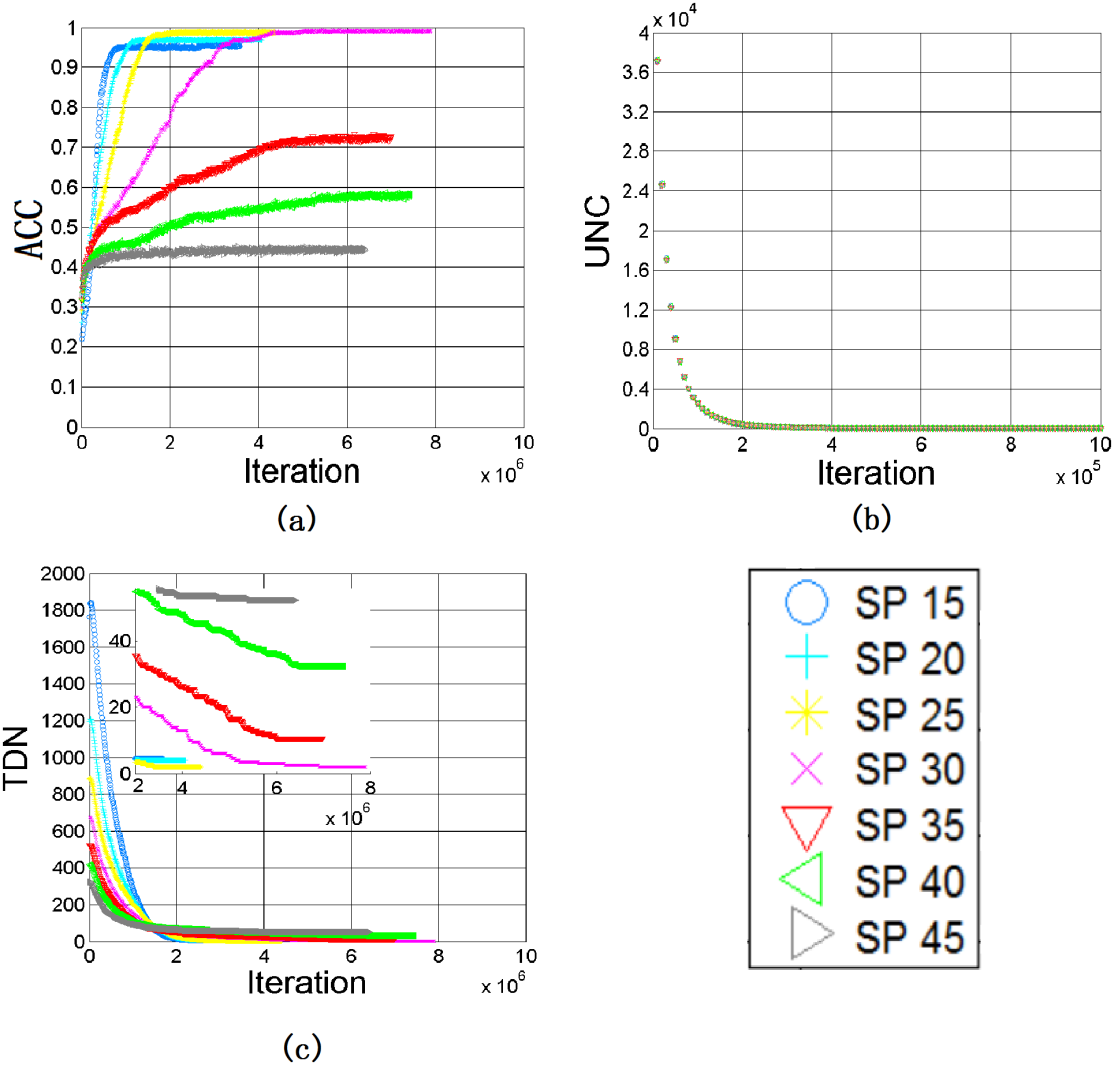
Results with *SS*_*T*_ = 10 and different values of *SP*_*T*_: (a) ACC, (b) UNC, (c) TDN.

We here study the impacts of subjective perception onto the learning performance. By keeping *SS*_*T*_ = 10, Fig 10(a), 10(b) and 10(c) depict the results of ACC, UNC and TDN against iterations, respectively, for different values of *SP*_*T*_.

When *SP*_*T*_ is small, say *SP*_*T*_ = 15, 20, 25 or 30, the final ACC improves with the increase of *SP*_*T*_, although the learning rate also becomes slower, as shown in Fig 10(a). The TDN in the population is also reduced from 4.6 to 1.6 in average, as given in Fig 10(c) and Table 3.

**Table 3:** ACC, TDN and UNC for different *SP*_*T*_ after 2 million iterations.

*SP*_*T*_	ACC	TDN	UNC
15	95.33%	4.6	0
20	96.97%	4.0	0
25	98.73%	2.0	0
30	99.30%	1.6	0
35	72.52%	10.5	0
40	58.15%	32.4	0
45	44.29%	52.4	0

**Fig 11:**
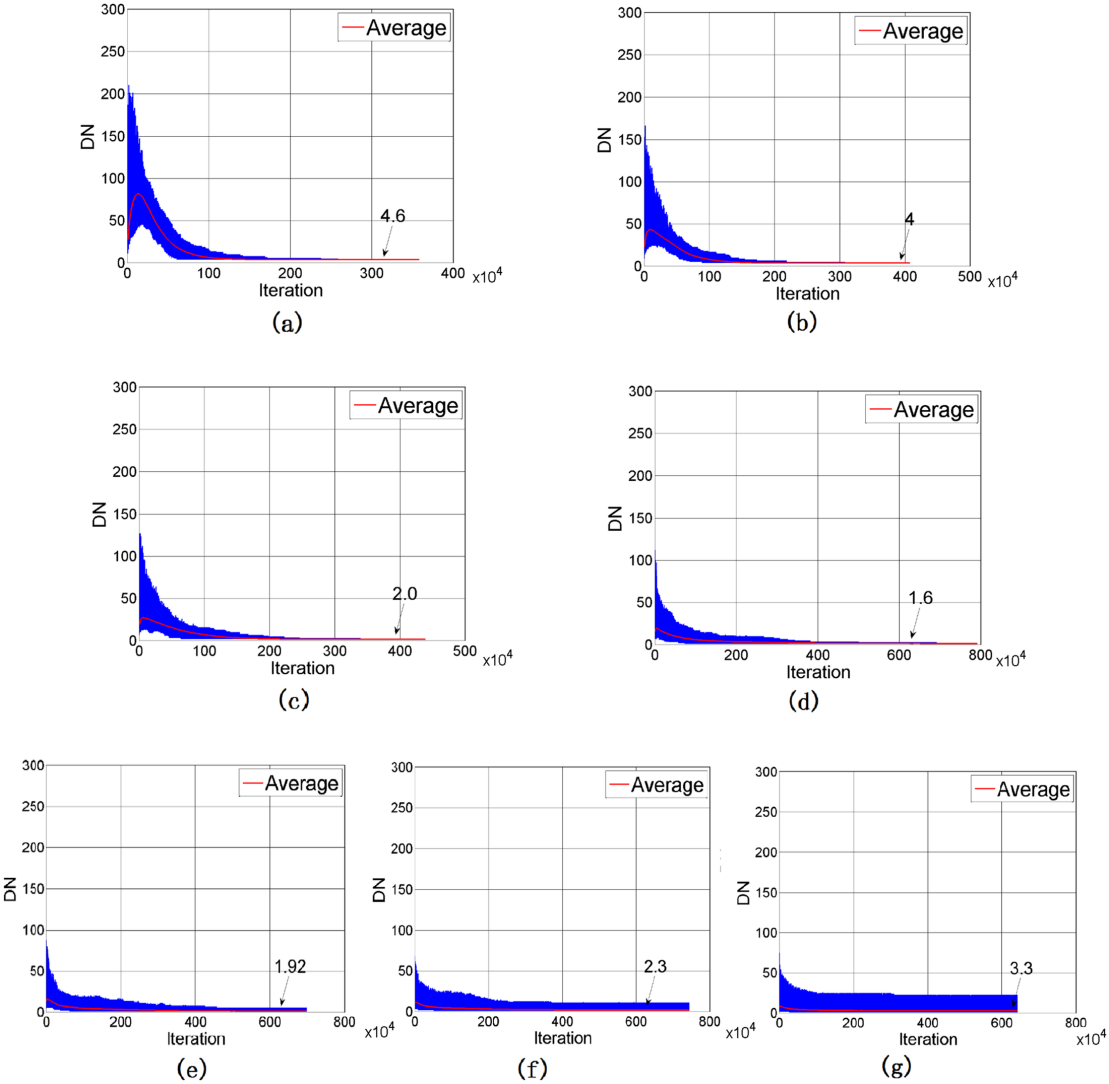
Number of different names in every agent with *SS*_*T*_ = 10 and different values of *SP*_*T*_: (a) *SP*_*T*_ = 15(b) *SP*_*T*_ = 20(c) *SP*_*T*_ = 25(d) *SP*_*T*_ = 30(e) *SP*_*T*_ = 35 (f) *SP*_*T*_ = 40 (g) *SP*_*T*_ = 45.

When *SP*_*T*_ and *SS*_*T*_ are comparable, the learning process is more effective. It is because the update of the name within *SS*_*T*_ from the other will easily lead to a real impact onto the color categorization of an agent. Moreover, for a large *SP*_*T*_, the influence of a color’s name become broadly, merging more color centroids to the same name. Consequently, it leads to the reduction of the number of DN in every agent and also the population, as shown in Fig 11(a)–11(d). The plots of UNC for different *SP*_*T*_, as revealed in Fig 10(b), are very similar. It is because UNC is mainly affected by *SS*_*T*_, which actually governs the name assignment to the color centroids.

**Fig 12:**
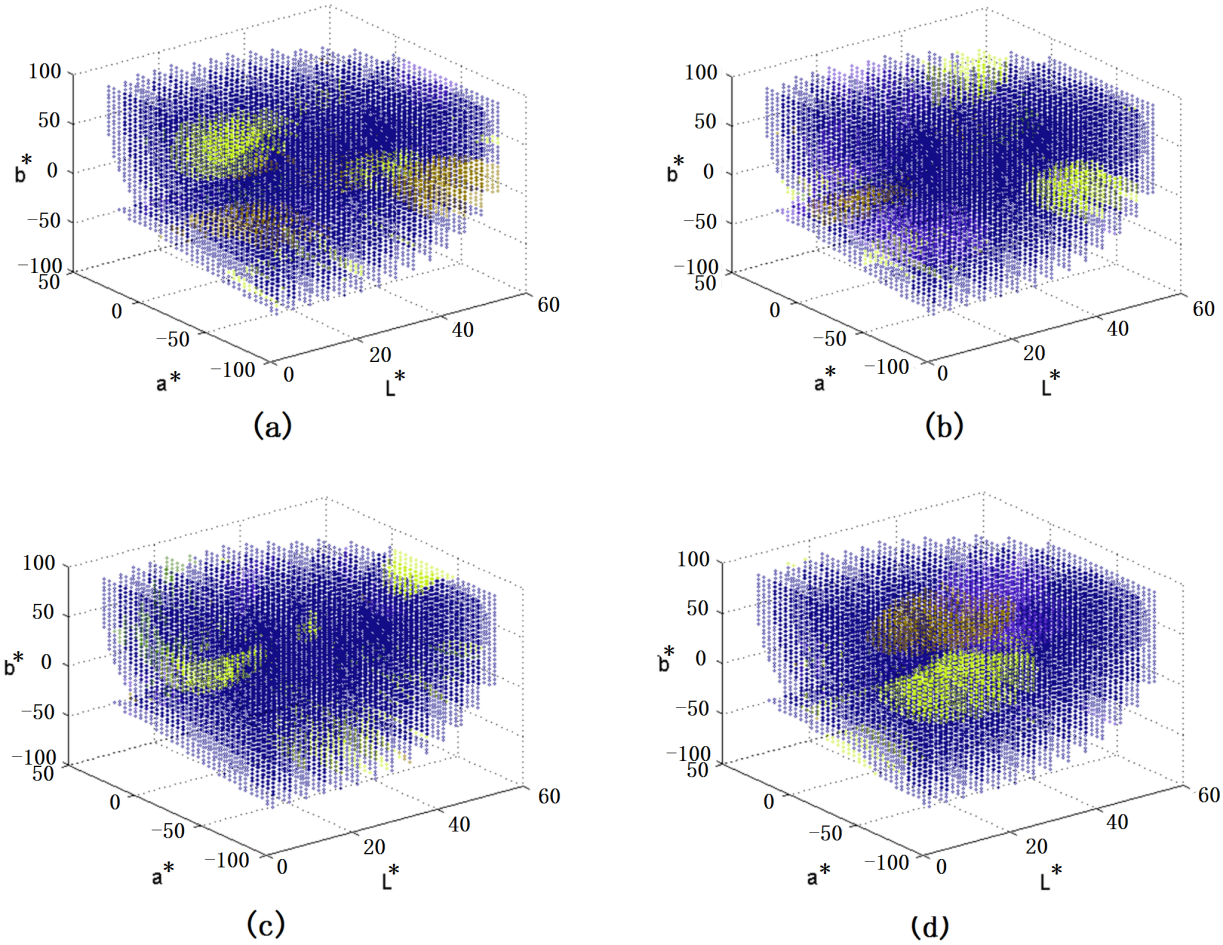
Name variation of agents with *SS*_*T*_ = 10 and *SP*_*T*_ = 40: (a) Iteration 1,500,000, (b) Iteration 2,000,000, (c) Iteration 2,500,000, (d) Iteration 3,000,000.

However, the ACC drops rapidly when *SP*_*T*_ is further increased from 30 to 45, as shown in Fig 10(a) and Table 3. From Fig 11(d)–11(f) and Table 3, a high variance is found in the names among agents. When *SP*_*T*_ = 45, ACC drops to 44.29% and TDN becomes 52.4, concluding that the game interaction becomes very inefficient for large *SP*_*T*_. Referring to the definitions of *SP*_*T*_ and *SS*_*T*_, the name of a picked color is determined by the majority rule over the set specified by *SP*_*T*_. Therefore, it is difficult to change one’s color categorization because, even though an agent tried to learn from the other, only a small subset as defined by *SS*_*T*_ is affected. This effect is clearly demonstrated in Fig 12. Here, we randomly select an agent from the population and monitor the names of its color centroids which have ΔE ≤ 40 with X = (14, -26, 26). As shown in Fig 12, since *SP*_*T*_ is large, many color centroids are included for the determination of the name of Color X. In the case with *SS*_*T*_ = 10 and *SP*_*T*_ = 40, there are about 15000 color centroids within SP but only about 500 color centroids within SS. Therefore, even though the agent could act as the Hearer and acquire correct name from the Speaker in a game, the majority remains wrong and the game will very likely fail again. Consequently, a low ACC is resulted for large *SP*_*T*_.

From the optimization point of view, it is important to have high ACC. It means that agents are to have similar color categorization, which is essential for cultural knowledge development in a population. On the other hand, in order to maintain a richness in color categorization, it is necessary to have as many different color categories as possible, each assigned to a name. It is similar to the practical case, when colors such as Alizarin crimson, Amaranth, American rose are observed, due to the lack of vocabulary, ordinary people may just name them all as Red.

## Discussions

In this work, a novel domain learning naming game (DLNG) model has been developed. In contrast to all the other existing models, DLNG allows categorization of a domain, which is essential for complex cognitive development. As illustrated, color categorization can be explored under DLNG. Moreover, a more intelligent agent has been designed to cope with domain learning in color categorization, supported by the definitions of subjective perception and subliminal stimulation.

As shown in the baseline results, four distinct colors are obtained, indicating that there is no sorites paradox. Further, the final four colors achieve a good consensus (high ACC) and a high color differentiation. That is close to the observation of some focal colors based on the study of color categorization and naming in linguistics. It should be pointed out that the debate between relativist and universalist in this issue has been lasted for a long time [39–42]. However, the impacts of learning processes in the society are usually ignored. Our study indicates that interactions within a society can indeed be a significant factor for the reaching of the focal colors.

**Fig 13:**
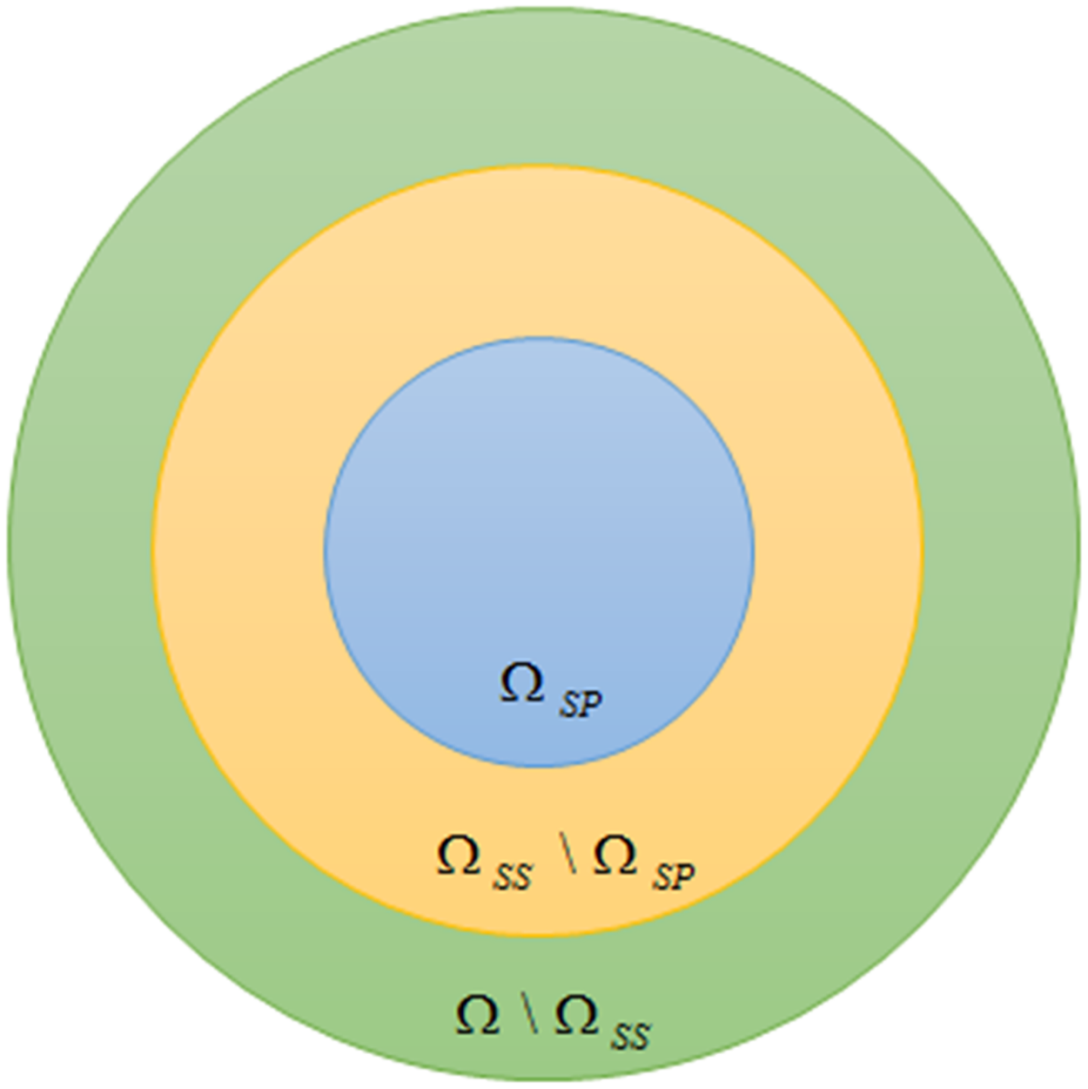
The sets defined by subjective perception and subliminal stimulation.

Furthermore, the definitions of subjective perception and subliminal stimulation allow the inclusion of uncertainty in the cognitive system of the agent. Referring to Fig 13, the colors within the inner circle are perceivably identical. The colors outside the outer circle are definitely another color. The region in-between reflects the uncertainty.

Our result shows that, if the ratio of uncertainty increases, i.e. (Ω_*SS*_ \ Ω_*SP*_): Ω_*SP*_ is larger, caused by the decrease of Ω_*SP*_ or increase of Ω_*SS*_, a decrease in the number of categories is noticed. Consequently, it improves the ACC, but it may not be good for the community due to the lack of vocabulary for communications. Further increase in uncertainty probably results in the collapse of the color categorization. On the other hand, if (Ω_*SS*_ \ Ω_*SP*_): Ω_*SP*_ is small, meaning that agents are very precise in color categorization without much flexibility (or uncertainty in other word), the learning process would be slow. A degradation of ACC can also be observed as it is relatively difficult to reach consensus.

## Supporting information

S1 Appendix Results for 100 agents. (PDF)

S1 File Adjacency matrix of 100 agents and 300 agents. (ZIP)
