# Supplementary material for: Domain learning naming game for color categorization
Source: PLoS One. 2017 Nov 14;12(11):e0188164. doi: 10.1371/journal.pone.0188164 (PMC5685623; doi:10.1371/journal.pone.0188164)
Supplement: S1 Appendix — (PDF) [file pone.0188164.s001.pdf]

S1 Appendix. Results for 100 agents

The domain learning naming game described in the manuscript has been applied for two cases, with the number of agents equal to 100 and 300, respectively.

The connectivity of agents is governed by an underlying topology, generated by the BA scale-free model with an average degree of 4. The corresponding adjacency matrix can be found in data in S2_File. The degree distributions of the nodes of these two topologies are shown in Fig. S1.

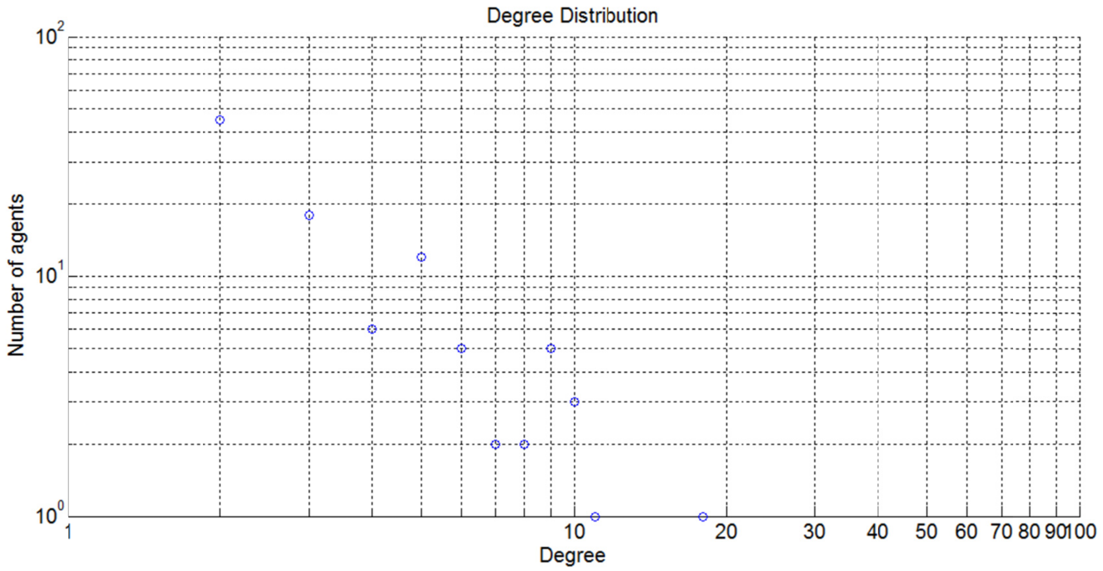

(a)

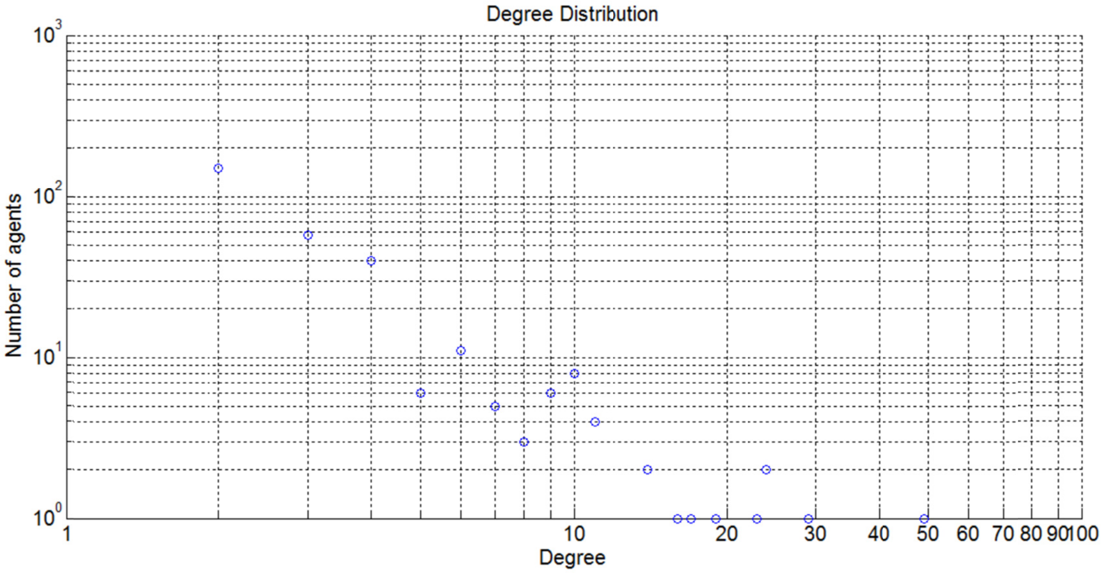

(b)

Fig S1. Degree distribution: (a) network of 100 agents (b) network of 300 agents

The results for network of 300 agents are given in the manuscript. The followings show the results for 100 agents. The results of both cases are very similar, and the descriptions in the manuscript are also applied for the case of 100 agents.

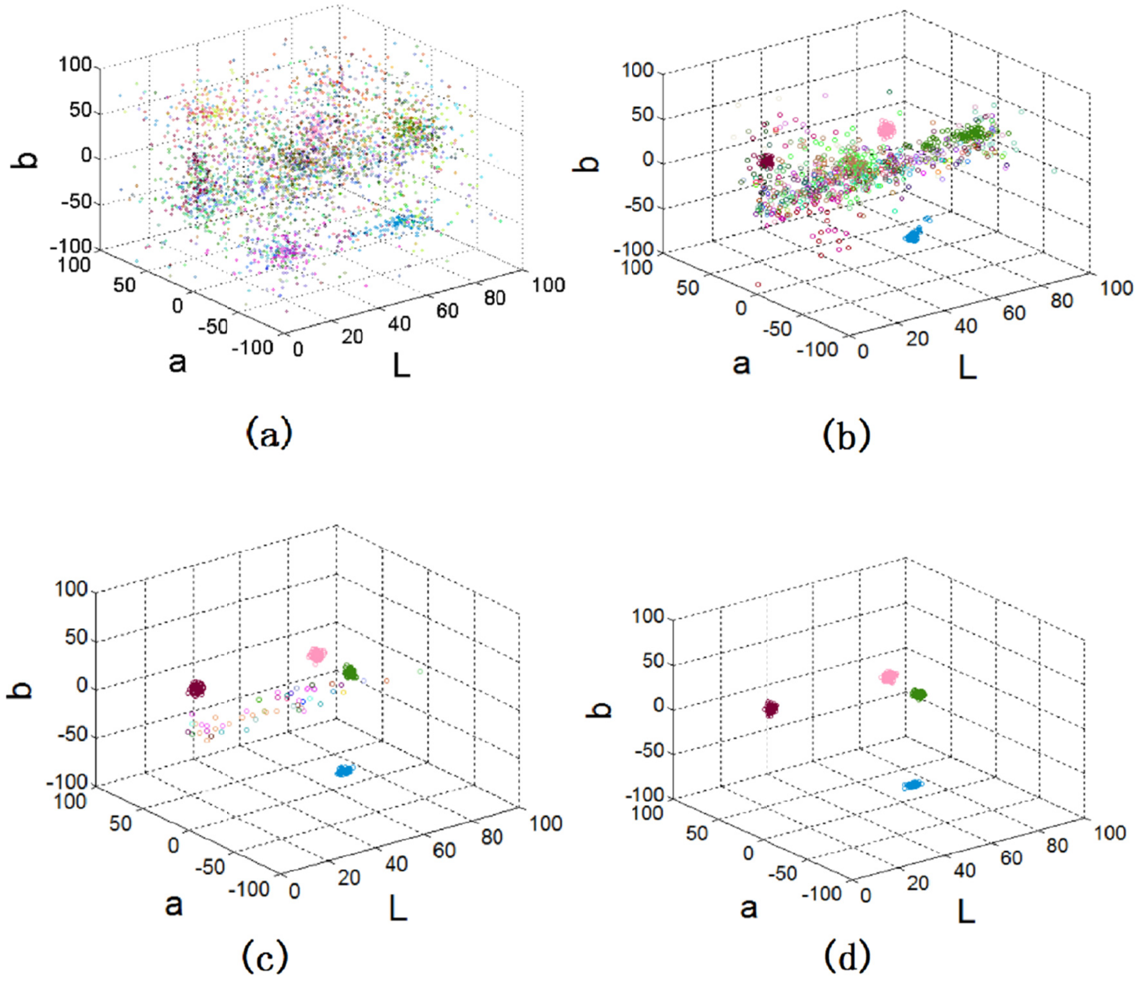

Fig S2. Variation of memory in the agents (a) after 10,000 games, (b) after 250,000 games, (c) after 750,000 games, (d) after 1,000,000 games.

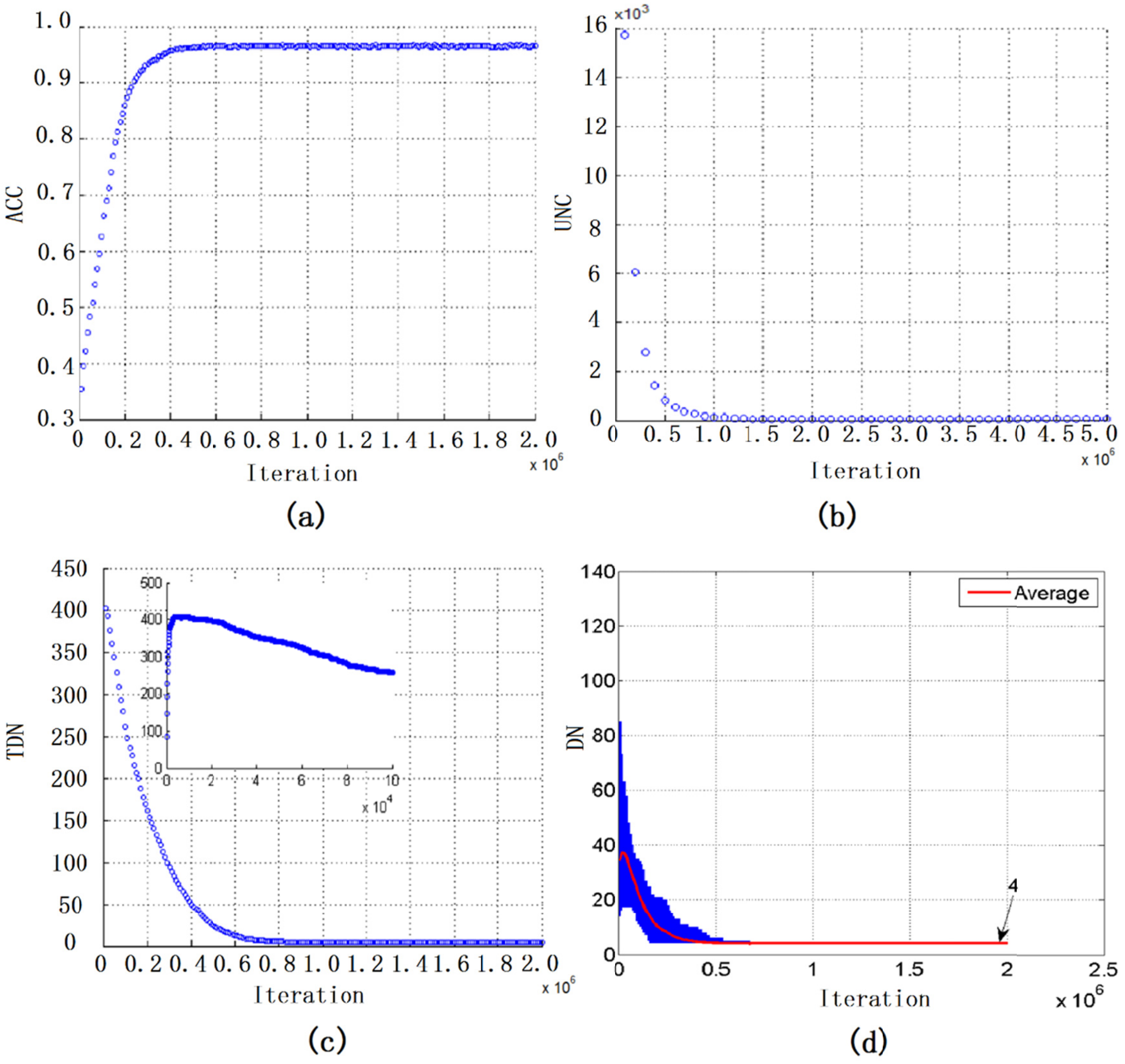

Fig S3. Simulation results of DLNG over a BA network of 100 agents with $SP_T = 20$ **and** $SS_T = 10$ **: (a) ACC, (b) UNC, (c) TDN, (d) DN.**

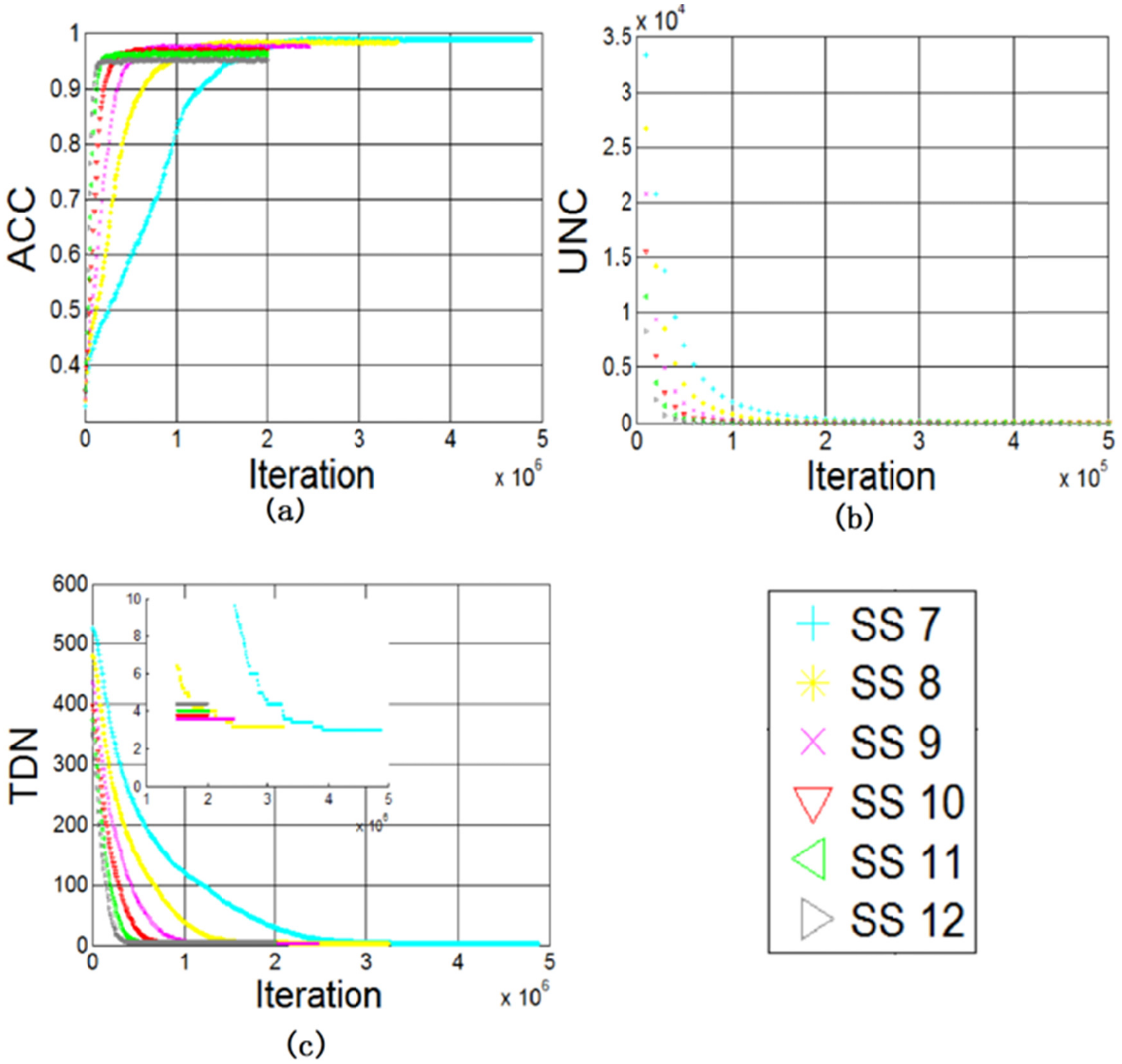

Fig S4. Results with $SP_T = 20$ and different values of SS_T: (a) ACC, (b) UNC, (c) TDN.

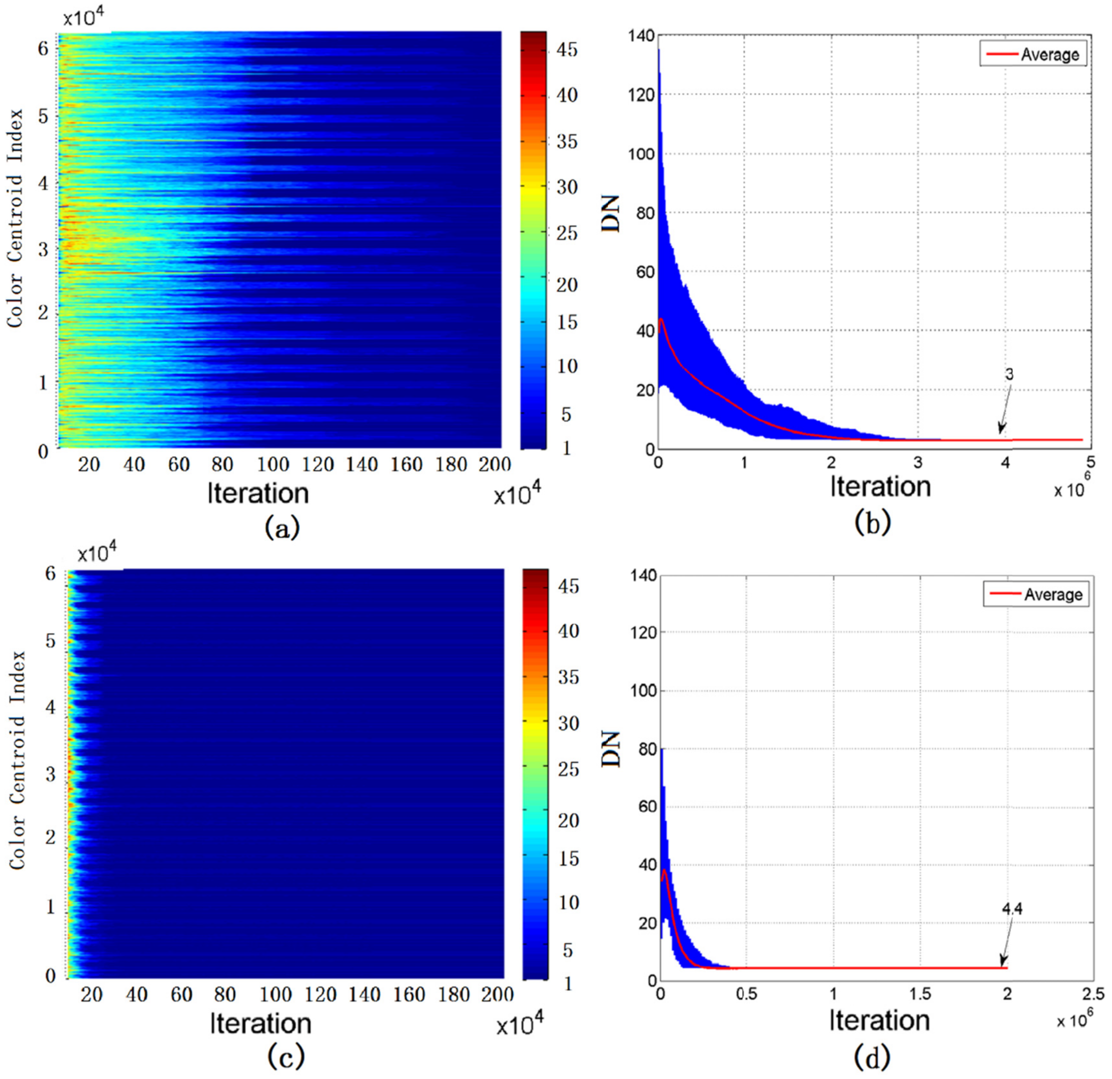

Fig S5. Evolution of the name variation for Ω: (a) and (b) for $SS_T = 7$, **(c) and (d) for** $SS_T = 12$.

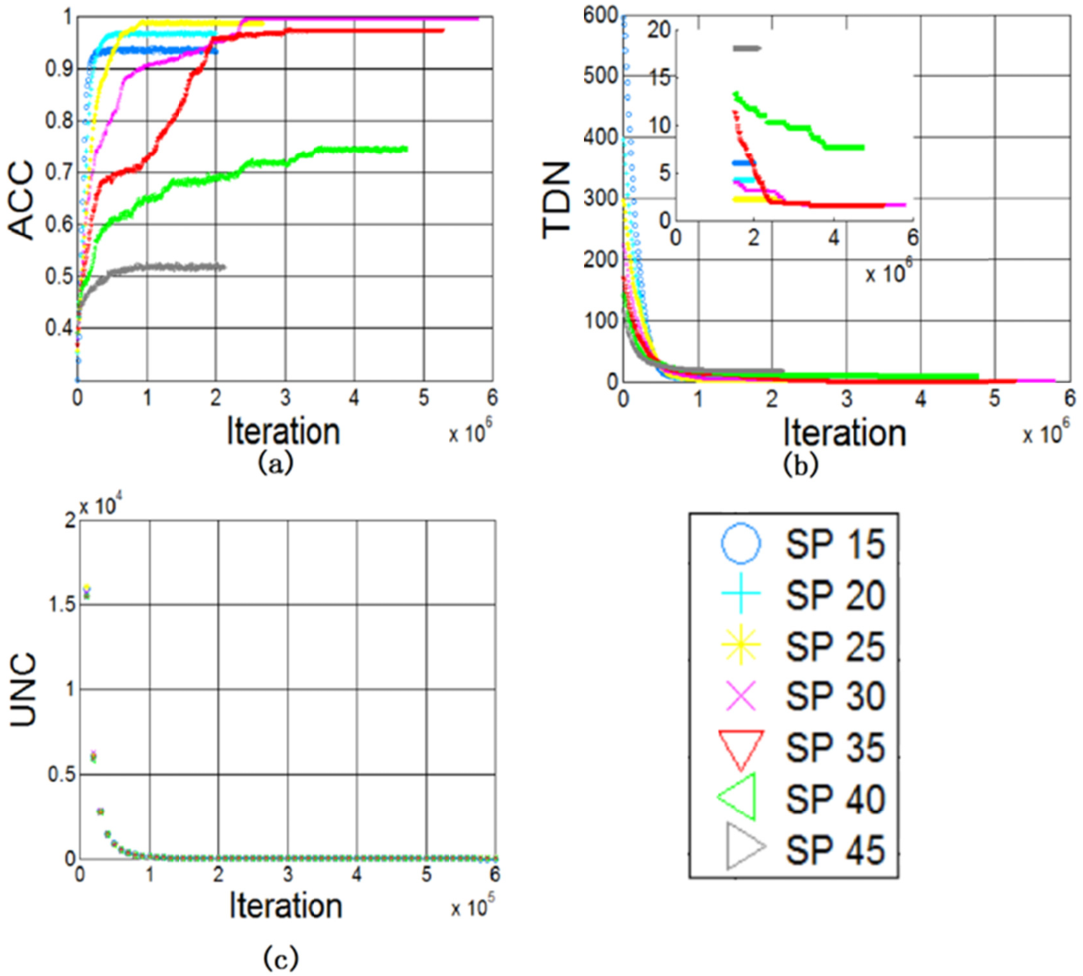

Fig S6. Results with $SS_T = 10$ and different values of SP_T: (a) ACC, (b) TDN, (c) UNC

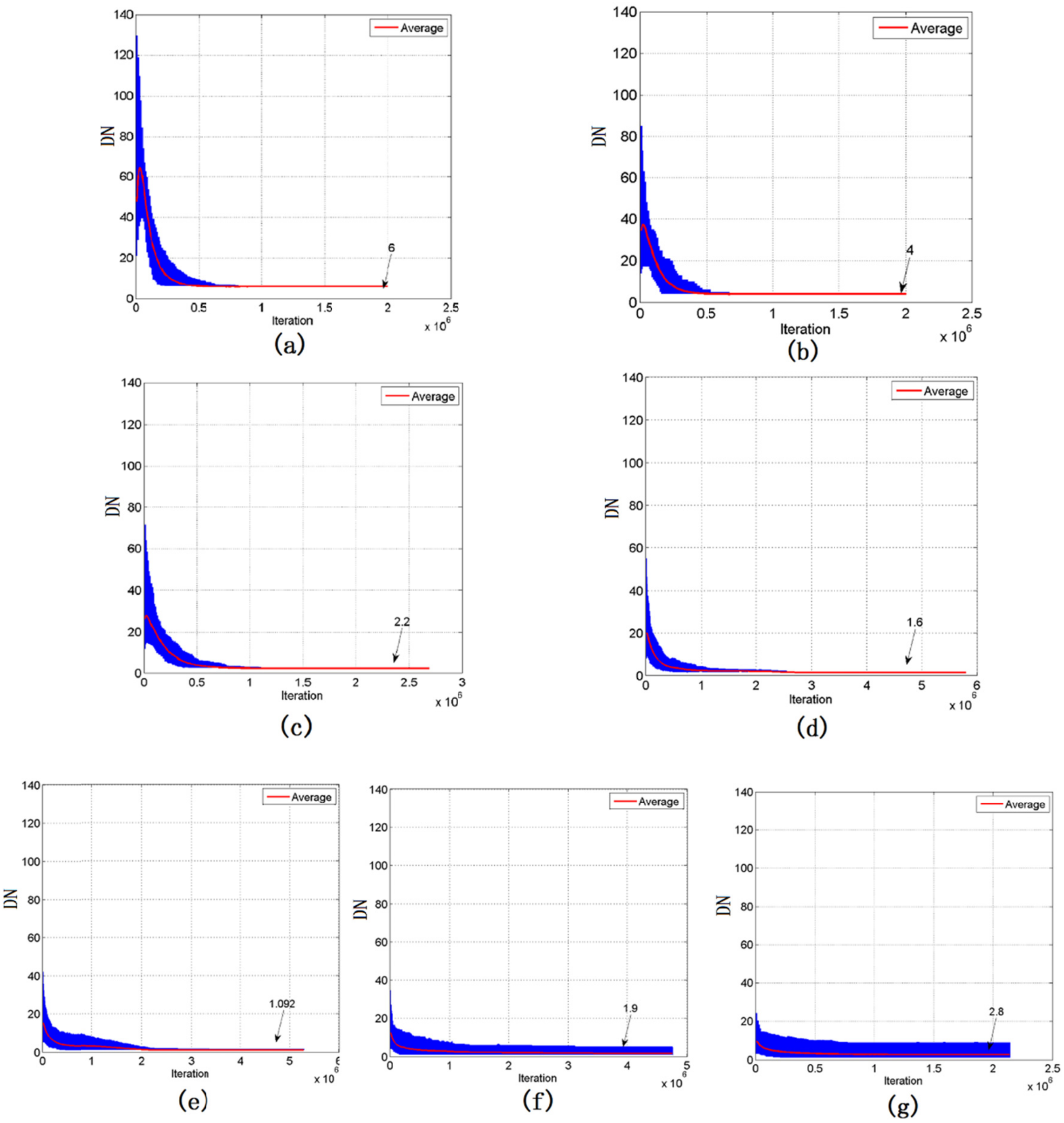

Fig S7. Number of different names in every agent with $SS_T = 10$ and different values of SP_T:
(a) $SP_T = 15$, **(b)** $SP_T = 20$, **(c)** $SP_T = 25$, **(d)** $SP_T = 30$, **(e)** $SP_T = 35$, **(f)** $SP_T = 40$, **(g)** $SP_T = 40$.
